# Effect of X-ray spot size on liquid jet photoelectron spectroscopy

**DOI:** 10.1107/S1600577515016306

**Published:** 2015-09-30

**Authors:** Giorgia Olivieri, Alok Goel, Armin Kleibert, Matthew A. Brown

**Affiliations:** a Laboratory for Surface Science and Technology, Department of Materials, ETH Zürich, CH-8093 Zurich, Switzerland; b Swiss Light Source, Paul Scherrer Institut, CH-5023 Villigen PSI, Switzerland

**Keywords:** near-ambient pressure photoemission, gas-phase photoemission, liquid–air interface

## Abstract

A 30 µm pinhole is introduced in the intermediate focus of the SIM beamline at the Swiss Light Source to improve the spot size at the second downstream focus, which is used here for liquid jet X-ray photoelectron spectroscopy experiments. The 30 µm pinhole reduces the beam dimensions from 250 (v) × 100 (h) µm to 75 × 45 µm for a vertical exit slit of 100 µm. The smaller X-ray spot results in a substantial decrease in the gas-phase contribution of the spectra from 40% down to 20% and will help to simplify the interpretation and peak assignments of future experiments.

## Abstract 2

A small pinhole is used to reduce the X-ray spot size for liquid jet photoelectron spectroscopy applications.

## 1. Introduction   

Liquid jet (LJ) based X-ray photoelectron spectroscopy (XPS) (Siegbahn & Siegbahn, 1973 ▸; Winter & Faubel, 2006 ▸) is a powerful analytical probe of electronic and geometric structures at liquid interfaces and has found widespread application over the last decade in diverse areas of environmental (Brown *et al.*, 2012 ▸, 2009 ▸), biological (Ottosson *et al.*, 2011 ▸), soft-matter (Brown *et al.*, 2014 ▸; Brown, Beloqui Redondo, Sterrer *et al.*, 2013 ▸) and fundamental (Björneholm *et al.*, 2014 ▸; Winter *et al.*, 2004 ▸) sciences. One often-limiting factor in the interpretation of the spectra is the overlap of the photoelectron lines originating from liquid with those originating from gas-phase molecules that surround the liquid jet in vacuum or at ambient pressures. This contribution can be exaggerated when the X-ray spot size greatly exceeds that of the liquid jet (typically <30 µm) and nearly suppressed when the two are of the same size. These overlapping peaks may lead to the incorrect assignment of the liquid peaks or prevent peak assignment altogether. The latter is often the case in the valence-band region where multiple peaks are present from both condensed and gas phases.

Modifying the focal X-ray spot profile of a synchrotron beamline can, in principle, be achieved using, for example, Kirkpatrick–Baez mirror pairs or Fresnel zone plates. These approaches require a dedicated beamline and the appropriate endstation design and are, therefore, not easily implemented into existing equipment. Here we demonstrate improved performance for LJ-XPS by introducing a 30 µm circular pinhole in the intermediate focus of the SIM beamline (Flechsig *et al.*, 2010 ▸) at the Swiss Light Source (SLS). In the normal operation conditions of the beamline, the pinhole reduces the spot size at the second refocusing station where the LJ-XPS is operated and results in spectra that have up to 20% lower gas-phase contributions. This approach is straightforward to install and works for all wavelengths. Moreover, it requires virtually no capital investment.

## 2. Experimental setup   

**Figure 1:**
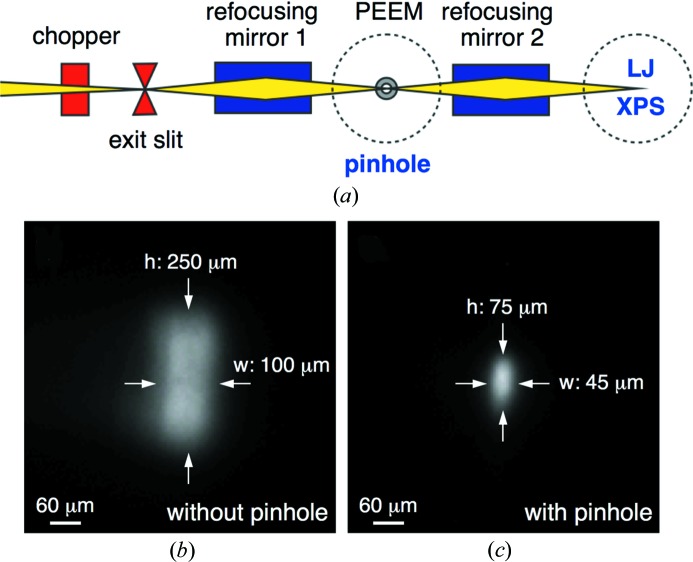
SIM beamline layout (*a*) and X-ray spot image after the second toroidal refocusing mirror: (*b*) without a pinhole and (*c*) with a 30 µm pinhole in the intermediate focus of the beamline. In both images the exit slit of the beamline is 100 µm and the photon energy is 800 eV.

The SIM beamline is equipped with two 3.8 m Apple II undulators in the X11M straight section of the SLS followed by a plane-grating monochromator (PGM) and employs a collimated light scheme (Follath & Senf, 1997 ▸) to provide an intermediate focus for a permanently installed photoemission electron microscope (PEEM). The latter is equipped with a sample manipulator that allows for accurate and stable positioning of the sample in both the focus of the microscope and the intermediate focal plane of the beamline (Le Guyader *et al.*, 2012 ▸). When retracting the PEEM sample a second re­focusing mirror further downstream provides a nearly 1:1 image of the intermediate focus (Flechsig *et al.*, 2010 ▸) for operation of a variety of exchangeable endstations (Fig. 1 ▸ *a*). Here, we mount a precision platinum pinhole (Plano GmbH) to an adapted PEEM sample holder and place the pinhole (instead of a PEEM sample) in the intermediate focus to modify the spot profile at the second focal plane. The effect of the pinhole on the X-ray spot profile in the second focal plane is monitored using a phosphor screen and a CCD camera. XPS measurements are performed using the near ambient pressure photoemission (NAPP) endstation of the SLS (Brown, Beloqui Redondo, Jordan *et al.*, 2013 ▸). A 19 µm liquid jet of 0.05 *M* NaCl is operated in vacuum (10^−4^ mbar). O 1*s* spectra are collected at ∼160 eV kinetic energy (KE) using a photon energy of 700 eV. Gas phase N_2_ spectra were recorded at 0.3 mbar using the same beamline settings as for the liquid jet experiments. A 200 nm-thick SiN_*x*_ window separates the ultrahigh vacuum of the beamline from the ambient N_2_ environment. All spectra were recorded at a pass energy of 50 eV with a 300 µm entrance slit on a Scienta R4000 HiPP-2 analyzer.

## 3. Results and discussion   

The X-ray spot profile after the second toroidal refocusing mirror is shown in Fig. 1 ▸(*b*) for a photon energy of 800 eV. With a vertical exit slit size (dispersive plane) of 100 µm we measure an X-ray spot size of 250 (h) × 100 (v) µm. Inserting a 30 µm pinhole at the intermediate focus of the beamline reduces the X-ray size to 75 (h) × 45 (v) µm (Fig. 1 ▸ *c*). Under these conditions the pinhole decreases the total flux at the second focal plane by ∼85%, as measured by an IRD SXUV 100 photodiode.

**Figure 2:**
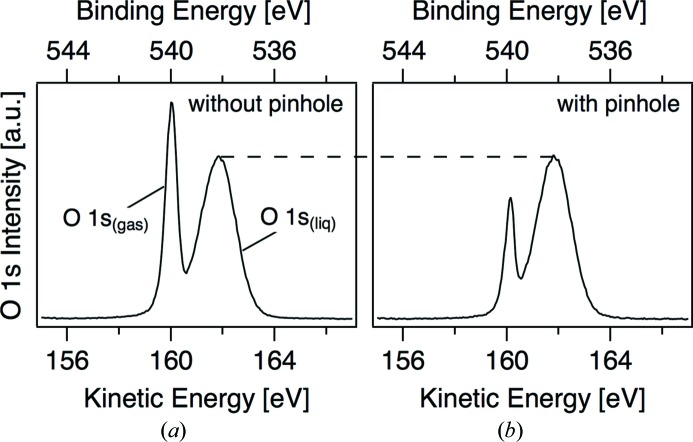
O 1*s* spectra from a 19 µm liquid jet of 0.05 *M* NaCl collected with a photon energy of 700 eV: (*a*) without a pinhole and (*b*) with a 30 µm pinhole in the intermediate focus of the beamline. Both spectra are acquired using a 10 µm exit slit on the beamline. The peaks corresponding to the liquid jet, O 1*s*_(liq)_, and from gas phase, O 1*s*_(gas)_, are labeled only in (*a*).

O 1*s* spectra from a 19 µm liquid jet of 0.05 *M* NaCl operating in vacuum (10^−4^ mbar) are shown in Fig. 2 ▸(*a*) (without pinhole) and Fig. 2 ▸(*b*) (with a 30 µm pinhole). The vertical exit slit of the beamline is 10 µm for these experiments and, therefore, any reduction in X-ray spot size is expected primarily in the horizontal (width) direction. Because the liquid jet flows in the vertical direction, the smaller X-ray width will lead to fewer gas-phase molecules being ionized between the liquid jet and the entrance aperture of the hemispherical analyzer. The spectra are deconvoluted into two components, O 1*s*_(gas)_ at low KE and O 1*s*_(liq)_ at high KE. Without the pinhole the integrated gas-phase area contributes 40% of the total signal, whereas with the 30 µm pinhole in the intermediate focus of the beamline the gas-phase signal is reduced by a factor of two (Table 1 ▸). It becomes immediately clear that introducing this small pinhole in the intermediate focus of the SIM beamline provides significant benefit for LJ-XPS experiments (or any experiment that will require a small X-ray focal size).

**Figure 3:**
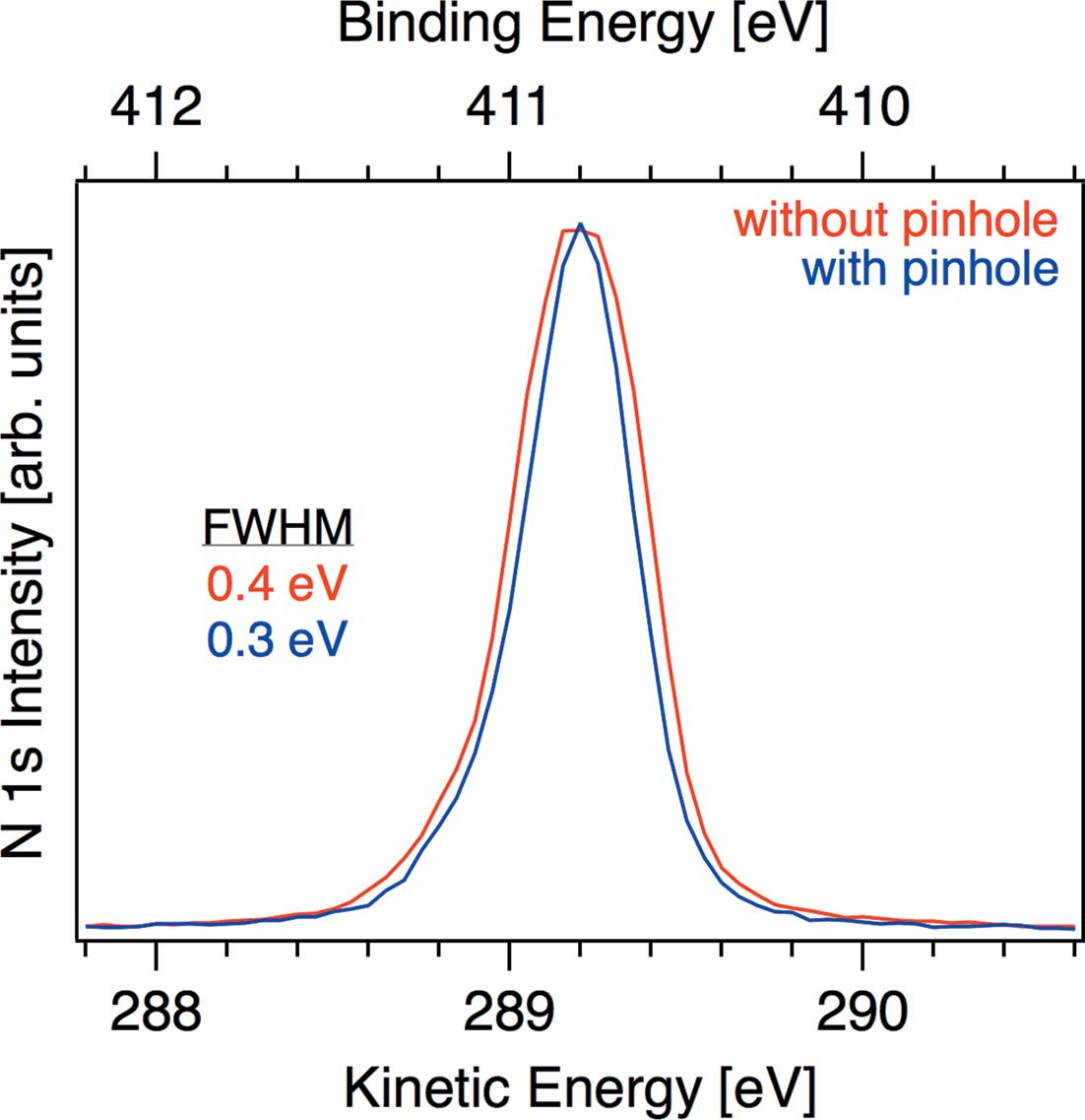
N 1*s* spectra from 0.3 mbar N_2(gas)_ collected with a photon energy of 700 eV: (red) without a pinhole and (blue) with a 30 µm pinhole in the intermediate focus of the beamline. Both spectra are acquired using a 10 µm exit slit on the beamline. The full widths at half-maximum (FWHM) are shown.

Further, we observe a small decrease in the FWHM of the O 1*s*_(gas)_ component when using the 30 µm pinhole (0.5 → 0.4 eV, Table 1 ▸). For these experiments the hemispherical energy analyzer operates at a theoretical resolution of 0.05 eV, suggesting that the decrease in FWHM originates from an increase in resolution of the beamline when using the 30 µm pinhole. To quantify this observation we performed an additional set of experiments in 0.3 mbar N_2_ (Fig. 3 ▸). The FWHM of the N 1*s* again shows a decrease when using the 30 µm pinhole (0.5 → 0.4 eV, Table 1 ▸) confirming that, in addition to reducing the X-ray spot size, the 30 µm pinhole increases the resolving power of the beamline. The origin of this behavior is a decrease in stray light of the beamline in its typical operating conditions (cutoff by the pinhole). However, because the FWHM of O 1*s*_(liq)_ is intrinsically broad, due to fluctuations in the local hydrogen-bonding environment of liquid water (Winter & Faubel, 2006 ▸), this improved resolution is only notable for gas-phase lines.

## 4. Conclusions   

A circular 30 µm pinhole introduced at an intermediate focus has been used to decrease the X-ray spot size at the second focal plane of the SIM beamline. This reduced the gas-phase contribution in LJ-XPS spectra by a factor of two and improved the energy resolution by about 25%. These improvements are easy to achieve and will prove useful for future experiments involving high vapor pressure organic solvents such as those employed in Li-ion batteries.

## Figures and Tables

**Table 1:** XPS peak fitting results

	Without a pinhole		With a 30 µm pinhole	
Peak	Contribution (%)	FWHM (eV)	Contribution (%)	FWHM (eV)
19 µm liquid jet of 0.05 *M* NaCl				
O 1*s*_(liq)_	59.9	1.5	79.0	1.5
O 1*s*_(gas)_	40.1	0.5	21.0	0.4

0.3 mbar N_2(gas)_				
N 1*s*_(gas)_	N/A	0.4	N/A	0.3
